# Development and validation of a prognostic model for assessing long COVID risk following Omicron wave—a large population-based cohort study

**DOI:** 10.1186/s12985-024-02400-3

**Published:** 2024-05-31

**Authors:** Lu-Cheng Fang, Xiao-Ping Ming, Wan-Yue Cai, Yi-Fan Hu, Bin Hao, Jiang-Hao Wu, Aikebaier Tuohuti, Xiong Chen

**Affiliations:** 1 Department of Otorhinolaryngology, Head and Neck Surgery, Zhongnan Hospital of Wuhan University, Wuhan, Hubei China; 2 Sleep medicine centre, Zhongnan Hospital of Wuhan University, Wuhan, Hubei China

**Keywords:** Long COVID, Omicron, Nomogram, Prediction

## Abstract

**Background:**

Long coronavirus disease (COVID) after COVID-19 infection is continuously threatening the health of people all over the world. Early prediction of the risk of Long COVID in hospitalized patients will help clinical management of COVID-19, but there is still no reliable and effective prediction model.

**Methods:**

A total of 1905 hospitalized patients with COVID-19 infection were included in this study, and their Long COVID status was followed up 4–8 weeks after discharge. Univariable and multivariable logistic regression analysis were used to determine the risk factors for Long COVID. Patients were randomly divided into a training cohort (70%) and a validation cohort (30%), and factors for constructing the model were screened using Lasso regression in the training cohort. Visualize the Long COVID risk prediction model using nomogram. Evaluate the performance of the model in the training and validation cohort using the area under the curve (AUC), calibration curve, and decision curve analysis (DCA).

**Results:**

A total of 657 patients (34.5%) reported that they had symptoms of long COVID. The most common symptoms were fatigue or muscle weakness (16.8%), followed by sleep difficulties (11.1%) and cough (9.5%). The risk prediction nomogram of age, diabetes, chronic kidney disease, vaccination status, procalcitonin, leukocytes, lymphocytes, interleukin-6 and D-dimer were included for early identification of high-risk patients with Long COVID. AUCs of the model in the training cohort and validation cohort are 0.762 and 0.713, respectively, demonstrating relatively high discrimination of the model. The calibration curve further substantiated the proximity of the nomogram’s predicted outcomes to the ideal curve, the consistency between the predicted outcomes and the actual outcomes, and the potential benefits for all patients as indicated by DCA. This observation was further validated in the validation cohort.

**Conclusions:**

We established a nomogram model to predict the long COVID risk of hospitalized patients with COVID-19, and proved its relatively good predictive performance. This model is helpful for the clinical management of long COVID.

**Supplementary Information:**

The online version contains supplementary material available at 10.1186/s12985-024-02400-3.

## Introduction

Long coronavirus disease (COVID), also known as Post acute sequelae of SARS-CoV-2 infection (PASC), is exerting a significant impact on a substantial global patient population comprising tens of millions who have contracted COVID-19 [1]. There are various definitions for long COVID, including the (1) persistent symptoms for more than 3 weeks after the initial onset of symptoms [2]; (2) persistent symptoms that appear during or after COVID-19 last more than 4 weeks [3]; or (3) continuation of signs and symptoms persisting beyond 12 weeks of infection [4]. long COVID has the potential to impact multiple organs or systems, giving rise to a diverse range of symptoms such as fatigue, sleep disturbances, dizziness, gastrointestinal symptoms, olfactory or gustatory impairments and chronic cough, etc [5].

Long COVID has been prevalent in various countries since the pandemic, and as the number of infected individuals continues to increase, an increasing number of individuals are troubled by long COVID [5, 6]. Long COVID undeniably presents substantial long-term risk to the physical, psychological, and social functioning of affected individuals, compelling numerous patients to experience job loss or reduced working hours, consequently leading to a notable decline in income [6, 7]. Certain sequelae exhibit a long course, presenting challenges in terms of treatment, high healthcare expenses, and imposing a substantial economic burden. Consequently, these sequelae pose a formidable challenge to both the social medical insurance system and the overall economy [8, 9].

In December 2022, China experienced a significant COVID-19 epidemic, resulting in infection rates exceeding 82% of the population within a two month [10]. The Chinese Center for Disease Control and Prevention’s data indicated that the main viral variant responsible for this transmission is Omicron [10]. Despite the comparatively lower severity and mortality rates associated with the Omicron variant in comparison to other variants, it does not mean that the risk of long COVID can be underestimated [11, 12]. The prevalence of the Omicron variant strain has resulted in a substantial increase in individuals infected, potentially exacerbating the incidence of long COVID and imposing a heavier economic and healthcare burden on society [11, 13]. Despite some comprehension regarding the clinical symptoms and therapeutic approaches for COVID-19, long COVID often presents with nonspecific symptoms, thereby lacking well-defined criteria for evaluating suspected long COVID patients and a definitive treatment regimen [5, 14, 15]. Therefore, better studying the potential risk indicators of long COVID holds paramount importance in the realms of public health and scientific research, as it facilitates the development of novel prevention and treatment measures.

This study aimed to investigate the risk factors associated with long COVID in hospitalized patients with COVID-19 during the Omicron epidemic in Wuhan from December 2022 to January 2023. Additionally, we endeavored to construct a nomograph model that can effectively predict the risk of long COVID. Compared to traditional prediction models, the nomograph is visualized and user-friendly, making it easier for clinical physicians to early assess the patient’s long COVID risk and implement appropriate monitoring and intervention, thereby potentially alleviating the patient’s related symptoms.

## Methods

### Study Population

This study retrospectively collected the data of patients who were diagnosed with COVID-19 infection and hospitalized in Zhongnan Hospital of Wuhan University from December 2022 to January 2023. All patients included in the study were confirmed as COVID-19 infection through three methods: (1) Nucleic acid test positive cases; (2) Those who test positive for antigens; (3) Patients identified by self-reported symptoms associated with COVID-19. All patients included in the study were clearly discharged from the hospital. According to the data from the Chinese Center for Disease Control and Prevention, the main type of virus variation in transmission is Omicron [10]. This study was approved by the Ethics Committee of Zhongnan Hospital of Wuhan University (No.2,023,083). The hospital ethics committee gave up the written informed consent of patients with COVID-19 infection.

### Data Collection

A comprehensive assessment of baseline population characteristics, comorbidities, and laboratory examination results at admission was conducted by extracting data from patients’ electronic medical records. Laboratory tests include blood tests (such as leukocytes, lymphocytes, neutrophils, etc.), inflammatory indicators (procalcitonin, interleukin 6), coagulation characteristics and cardiac enzymes. To mitigate the potential influence of sampling bias, data acquisition involved active communication with medical personnel and diligent verification of the collected information. The exclusion criteria for this study population are: (1) Pregnant patients; (2) Patients who have undergone surgery recently; (3) Patients with cognitive impairment; (4) Patients with chronic respiratory disease; (5) Patients who died during hospitalization. Six professionally trained researchers conducted a follow-up survey on the occurrence of long COVID among the population screened by the above criteria. Through the utilization of telephone, SMS, and on-site inquiry methods, patients were questioned regarding the presence of long COVID symptoms (such as fatigue, cough, sleeping difficulty, etc.) during the period of 4–8 weeks following their discharge. All enrolled patients were randomly divided into a training cohort and a validation cohort in a ratio of 7:3. According to the baseline characteristics, comorbidities, the results of the first laboratory examination at admission and the occurrence of long COVID, the nomogram model for predicting long COVID risk were constructed in the training cohort, and then verified in the validation cohort.

### Statistical analysis

Measurement data conforming to normal distribution were described as mean ± standard deviation (SD), measurement data with skewed distribution were described as median and interquartile range (IQR), and counting data were expressed as frequency and percentage (%). To compare the difference between groups, chi-square test was used for categorical variables and Wilcoxon rank-sum test was used for continuous variables. Univariable and multivariable logistic regression analysis were used to analyze the potential risk factors affecting the occurrence of long COVID. In order to build the prediction model of long COVID risk, the least absolute shrinkage and selection operator (LASSO) regularization algorithm with 10-fold cross-validation was performed to select potential predictors with non-zero coefficients in the training cohort [16, 17]. Factors selected in LASSO regression were further analyzed in a multivariate logistics model to identify the significant prognostic factors associated with long COVID risk, and a nomogram was used to visualize the model. The receiver operating characteristic (ROC) curve, area under the ROC curve (AUC) and calibration curve were used to evaluate the performance of the model, while the decision curve analysis (DCA) was used to determine the net income threshold of the prediction model. All the above analyses were conducted using R software (version 4.0.2), and in each statistical analysis, P-values < 0.05 were considered statistically significant.

## Results

### Characteristics of patients

**Fig. 1:**
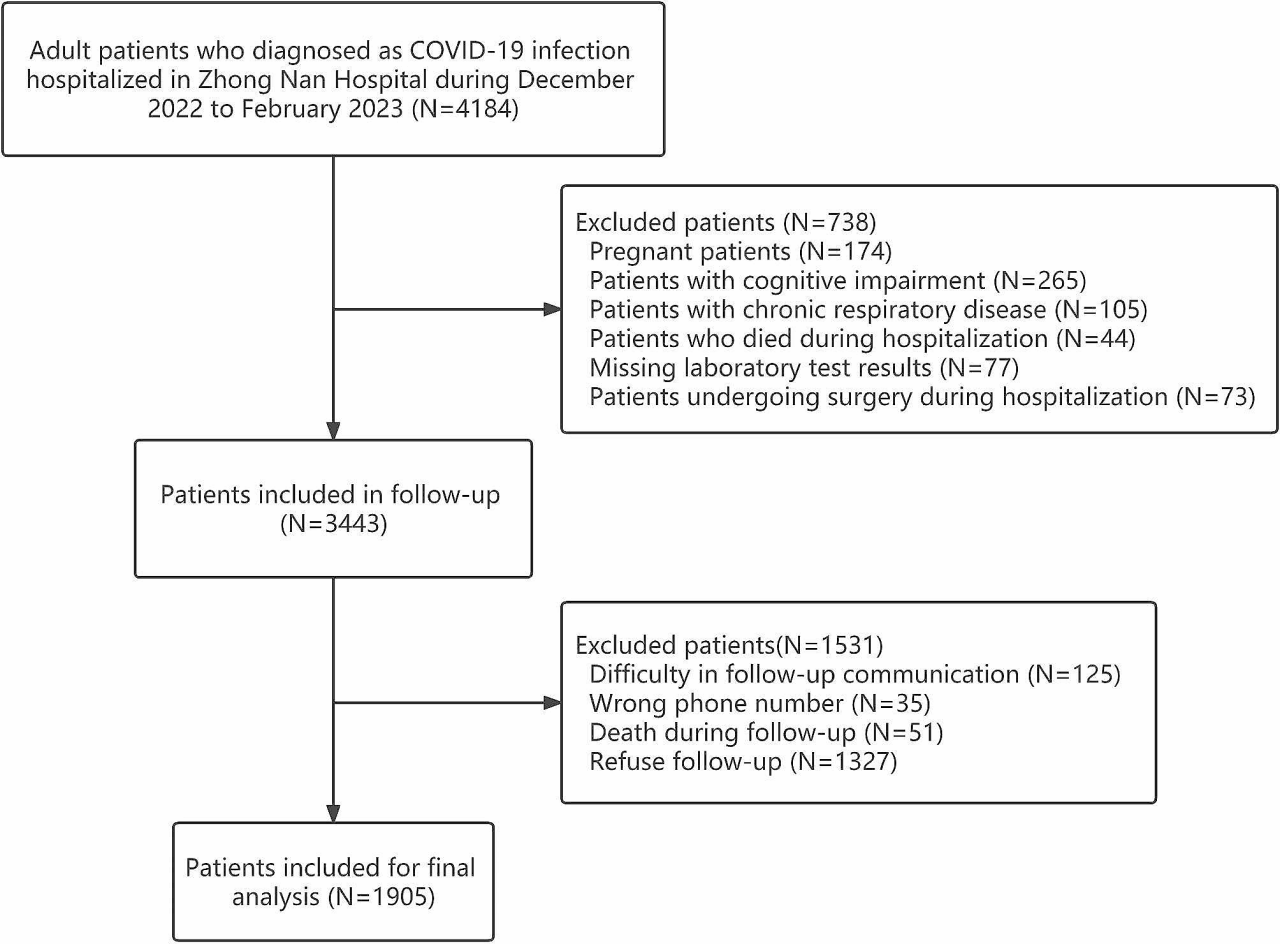
Flow chart showing the selection of study participants

After a meticulous selection process, a total of 1905 patients hospitalized for COVID-19 infection were finally included in the study. The detailed screening process is shown in Fig. 1. Table 1 shows detailed information on the baseline characteristics of these selected patients. The median age of the study participants was 58 years, with a minimum age of 18 years and a maximum age of 75 years. 1138 patients (59.7%) were male. Hypertension (31.3%) is the most common comorbidity. In addition, 1250 patients (65.6%) had more than two comorbidities. According to the vaccination status, unvaccinated individuals account for the majority (46.5%), followed by fully vaccinated individuals (35.7%), booster recipients (9.7%), and partially vaccinated individuals (8.1%). A total of 657 patients (34.5%) reported that they had symptoms of long COVID. The most common symptoms were fatigue or muscle weakness (16.8%), followed by sleep difficulties (11.1%) and cough (9.5%). Besides, the statistical results of the first laboratory examination during hospitalization are shown in Supplementary Table S1.

**Table 1:** Baseline characteristics of patients

Characteristics	All patients (N = 1905)	Training Cohort (N = 1333)	Validation Cohort (N = 572)	P-value
Gender				0.624
male	1138 (59.7%)	791 (59.3%)	347 (60.7%)	
female	767 (40.3%)	542 (40.7%)	225 (39.3%)	
Age (years), median (IQR)	58 (32,67)	58 (32,68)	58 (33,67)	0.529
Age group (years)				0.872
< 40	192 (10.1%)	136 (10.2%)	58 (10.1%)	
40 ~ 60	617 (32.4%)	428 (32.1%)	187 (32.7%)	
> 60	1096 (57.5%)	769 (57.7%)	327 (57.2%)	
Number of participants who self-reported long COVID symptoms				0.809
Long COVID	657 (34.5%)	462 (34.7%)	195 (34.1%)	
No symptoms of long COVID	1248 (65.5%)	871 (65.3%)	377 (65.9%)	
Most common self-reported long COVID symptoms				
Fatigue or muscle weakness	320 (16.8%)	222 (16.7%)	98 (17.1%)	0.721
Sleep difficulty	212 (11.1%)	150 (11.2%)	62 (10.8%)	0.065
Cough	181 (9.5%)	124 (9.3%)	57 (9.9%)	0.082
Smell disorder	105 (5.5%)	74 (5.6%)	31 (5.4%)	0.341
Decreased appetite	82 (4.3%)	57 (4.3%)	25 (4.4%)	0.264
Chest pain	40 (2.1%)	28 (2.1%)	12 (2.1%)	0.177
Skin rash	33 (1.7%)	23 (1.7%)	10 (1.7%)	0.637
Irregular fever	17 (0.9%)	12 (0.9%)	5 (0.9%)	0.324
Comorbidities				
Hypertension	592 (31.1%)	414 (31.1%)	178 (31.1%)	0.972
Diabetes mellitus	378 (19.8%)	264 (19.8%)	114 (19.9%)	0.909
Chronic kidney disease	323 (17.0%)	218 (16.4%)	105 (18.4%)	0.316
Chronic liver disease	260 (13.6%)	174 (13.1%)	86 (15.0%)	0.279
Rheumatic disease	41 (2.2%)	35 (2.6%)	6 (1.0%)	0.045
Neurological disease	95 (5.0%)	66 (5.0%)	29 (5.1%)	0.913
Cardiovascular disease	527 (27.7%)	386 (29.0%)	141 (24.7%)	0.061
Malignancy	373 (19.6%)	268 (20.1%)	105 (18.4%)	0.413
Number of comorbidities				0.897
0 ~ 1	655 (34.4%)	461 (34.6%)	194 (33.9%)	
2	1250 (65.6%)	872 (65.4%)	378 (66.1%)	
Vaccination status				0.621
Unvaccinated	886 (46.5%)	628 (47.1%)	258 (45.1%)	
Partially vaccinated	155 (8.1%)	103 (7.7%)	52 (9.1%)	
Fully vaccinated	680 (35.7%)	470 (35.3%)	210 (36.7%)	
Booster	184 (9.7%)	132 (9.9%)	52 (9.1%)	
Clinical severity				0.346
Nonsevere	1537 (80.7%)	1087 (81.5%)	450 (78.7%)	
Severe	338 (17.7%)	226 (17.0%)	112 (19.6%)	
Critical	30 (1.6%)	20 (1.5%)	10 (1.7%)	

### Univariable and multivariable logistic regression analysis

To ascertain the risk factors associated with the incidence of long COVID, we performed univariable and multivariable logistic regression analysis. In univariate analysis, significantly related risk factors include age, diabetes, chronic kidney disease, chronic liver disease, vaccination status, creatine kinase-MB, interleukin-6, procalcitonin, leukocytes, hematocrit, lymphocytes, eosinophil, hemoglobin, platelets, D-dimer, activated partial thromboplastin time, thrombin time, prothrombin time, and prothrombin time activity. In multivariate analysis, the significantly related risk factors included age, diabetes, chronic kidney disease, vaccination status, interleukin-6, procalcitonin, leukocytes, platelets, D-dimer, and activated partial thromboplastin time. Detailed information is available in Supplementary Table S2.

### Feature predictor selection and model evaluation

**Fig. 2:**
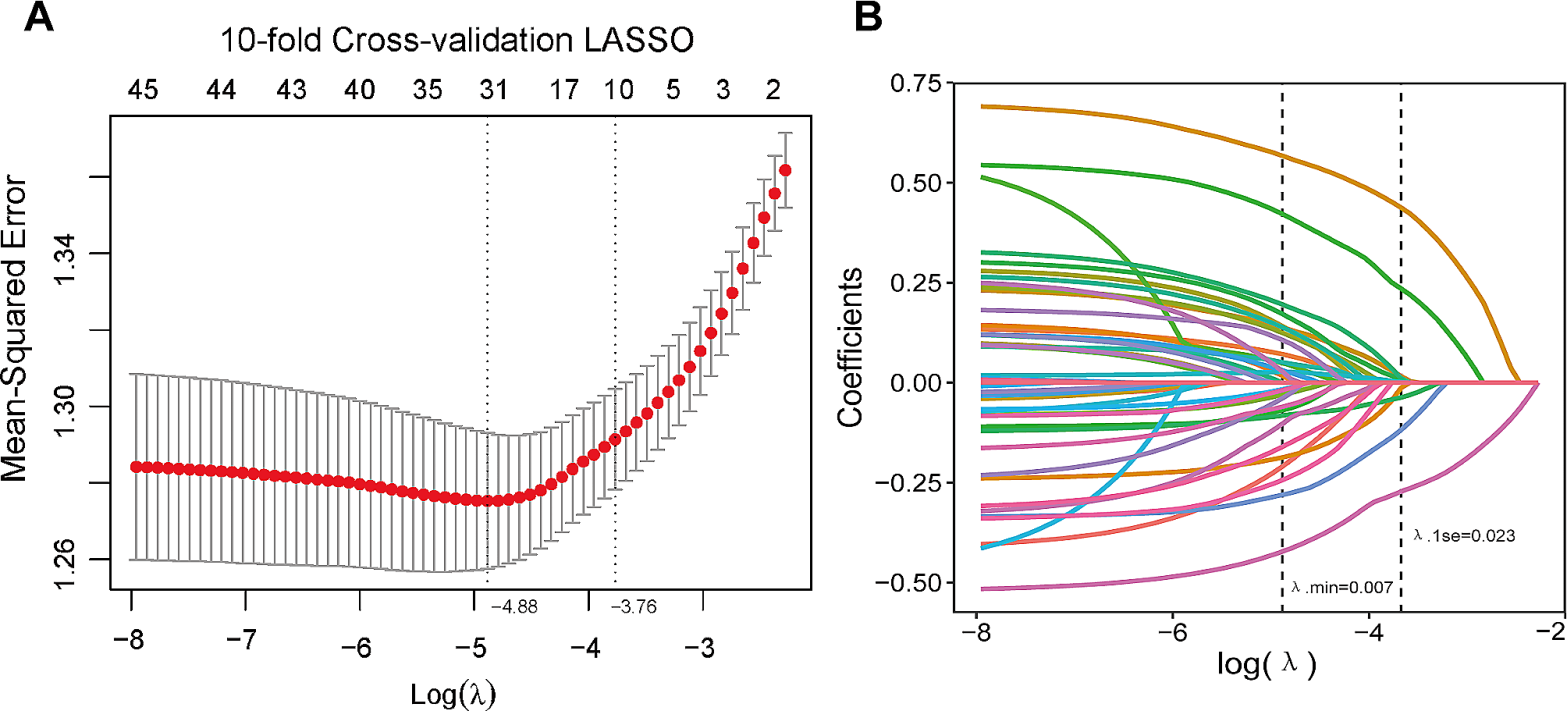
Feature variable selection using LASSO regression in the training cohort. (**A**) Tuning parameter selection cross-validation error curve. (**B**) Plot of the LASSO coefficient profiles

To facilitate the selection of variables for constructing a risk prediction model for long COVID, we randomly divided the research population into training cohort and validation cohort according to a ratio of 7:3. LASSO regression analysis was conducted on the training cohort, resulting in the identification of 9 potential predictive factors. As shown in Fig. 2, the most regularized and optimal model includes 9 variables, and its cross-validation error is within minimal standard error.

**Fig. 3:**
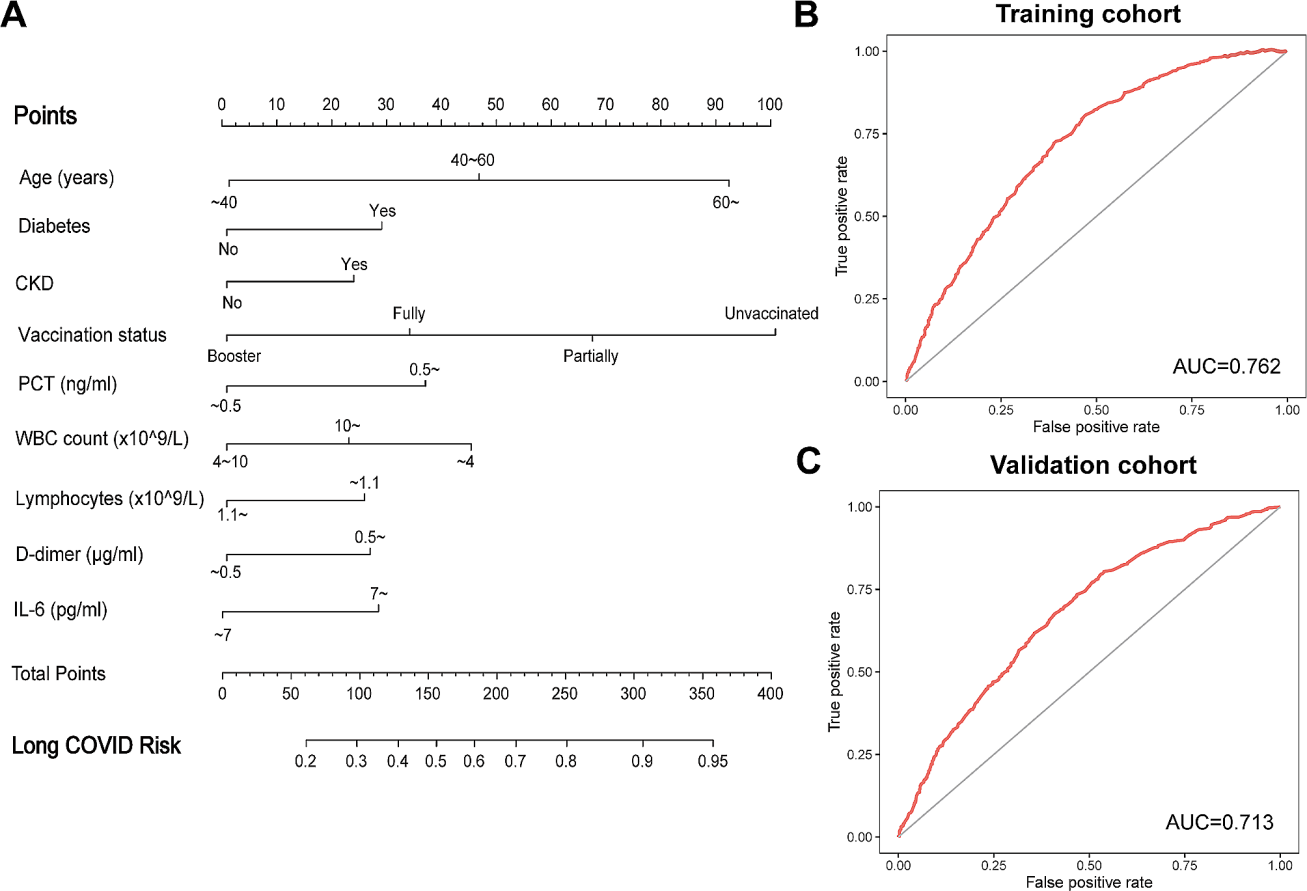
Development and application of the model for predicting the risk of long COVID. (**A**) The final nomogram prediction model. (**B**-**C**) Receiving operating characteristic curves showing the performance of the prediction model in predicting long COVID in the (**B**) training cohort and (**C**) validation cohort. Abbreviations: CKD, chronic kidney disease; PCT, Procalcitonin; WBC, white blood cell; IL-6, interleukin-6

**Fig. 4:**
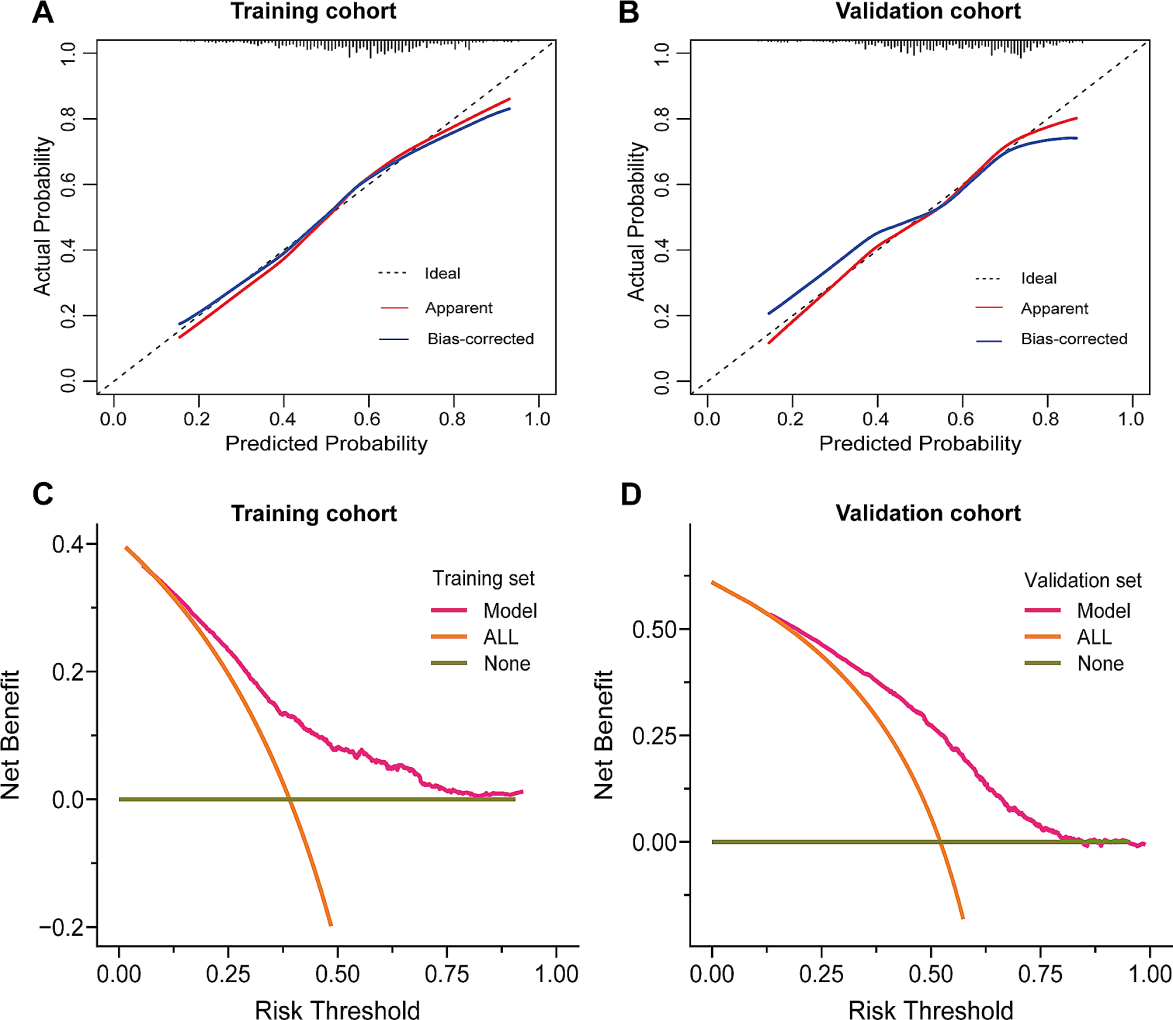
(**A**-**B**) Calibration curves for testing the stability of prediction model in the (**A**) training cohort and (**B**) validation cohort. (**C**-**D**) Decision curve analysis of prediction model in the (**C**) training cohort and (**D**) validation cohort

The final predictors included in the model were age, diabetes, chronic kidney disease, vaccination status, interleukin-6, procalcitonin, leukocytes, lymphocytes, and D-dimer. The OR and 95% CI of the above predictive factors are shown in Table 2. The above nine prediction factors were finally integrated into the risk prediction model of long COVID (Fig. 3A). As shown in Fig. 3B-C, the AUC of the model in the training cohort and validation cohort are 0.762 and 0.713, respectively, demonstrating relatively high discrimination of the model. What’s more, calibration curves show good consistency between the predicted and observed results in the training and validation cohort (Fig. 4A-B). The DCA curve is shown in Fig. 4C-D. In the training cohort, employing the predictive model to guide clinical intervention yields greater benefits compared to full patient intervention or no intervention when the patient’s long COVID risk threshold probability exceeds 10% (Fig. 4C). Similarly, in the validation cohort, utilizing the predictive model for clinical intervention is more advantageous than full patient intervention or no intervention when the probability of long COVID risk threshold for patients surpasses 15% (Fig. 4D). The DCA curve indicates that the nomogram model has good clinical net benefits.

**Table 2:** Coefficients, odds ratio, and 95% CIs in the final model

		Coefficients	Odds ratio (95% CI)	*p* - Value
Age (years)	< 40		Reference	
	40 ~ 59	0.74	2.10(1.64–2.71)	< 0.001
	> 60	1.13	3.09(1.92–15.68)	< 0.001
Diabetes mellitus	No		Reference	
	Yes	0.61	1.83(1.64–2.14)	0.005
Chronic kidney disease	No		Reference	
	Yes	0.51	1.66(1.42–1.92)	0.012
Vaccination status	Booster		Reference	
	Fully	0.65	1.91(1.25–2.81)	0.003
	Partially vaccinated	1.01	2.76(1.53–4.74)	< 0.001
	Unvaccinated	1.24	3.45(1.93–5.90)	< 0.001
IL-6 (pg/ml)	7		Reference	0.006
	> 7	0.57	1.76(1.11–2.01)	0.003
PCT (ng/ml)	< 0.05		Reference	
	> 0.05	0.67	1.95(1.20–2.98)	0.004
WBC(10^9/L)	4 ~ 10		Reference	
	> 10	0.49	1.63(1.44–1.88)	0.025
	< 4	0.72	2.05(1.63–2.57)	0.001
Lymphocyte (10^9/L)	> 1.1		Reference	
	< 1.1	0.53	1.69(1.22–1.93)	< 0.001
D-dimer (ug/ml)	< 0.5		Reference	
	0.5	0.55	1.73(1.44–2.20)	< 0.001

*Abbreviations* IL-6, interleukin 6; PCT, Procalcitonin; WBC, white blood cell

## Discussion

Previous studies have demonstrated that certain clinical indicators can be used to predict the severity and prognosis of patients with COVID-19 [18–20]. In our study, LASSO regression and logistic regression analysis were used to identify important factors associated with the occurrence of long COVID. Age, history of diabetes, history of chronic kidney disease, vaccination status, interleukin-6, procalcitonin, leukocytes, lymphocytes, and D-dimer were identified as predictors and used to develop a nomogram model to predict long COVID risk. Based on the evaluation of AUC values, calibration curves, and DCA, this nomogram model exhibits good discrimination and calibration in predicting long COVID risk, indicating its good performance and high clinical application value. Consequently, this tool can aid clinicians in resource optimization and the implementation of preventive measures.

This study comprised a cohort of 1905 patients who were hospitalized due to COVID-19 infection. 660 patients reported experiencing long COVID during the follow-up period, resulting in an incidence rate of 34.5%. It is important to note that there are currently no other studies conducted in China that have reported the incidence of long COVID specifically after the emergence of the Omicron variant, making direct comparisons challenging. However, research reports from the UK indicated that out of 56,003 Omicron cases, 2501 individuals (4.5%) experienced long COVID, whereas out of 41,361 Delta cases, 4469 individuals (10.8%) reported long COVID [12]. Furthermore, a separate study conducted in India revealed that 8.2% of 524 Omicron cases self-reported long COVID symptoms [21]. We speculate that the high incidence of long COVID in China may be due to the lack of antibodies against other COVID-19 strains.

In our study, the predominant symptoms reported by individuals with long COVID were fatigue or muscle weakness, followed by sleep difficulty and cough. This discovery aligns with prior scholarly literature, which consistently identified fatigue as the prevailing symptom of long COVID, exhibiting a prevalence rate exceeding 50% in certain investigations [22, 23]. Nevertheless, among children and young adults, emotional fluctuations emerge as the primary clinical manifestation, succeeded by fatigue [24]. Furthermore, in addition to fatigue, various symptoms including cough, chest pain, breathing difficulties, and muscle pain have also been widely reported [6, 25]. However, it is imperative to acknowledge that long COVID exhibits non-specific symptoms, and many symptoms are closely related to the patient’s comorbidities. Consequently, these non-specific symptoms may lead to deviation during follow-up, necessitating further research to elucidate the correlation between these symptoms and long COVID.

In our prediction model, the risk of long COVID in elderly and infirm patients (such as patients aged ≥ 60 years, with diabetes and chronic kidney disease) is significantly increased. Consequently, healthcare professionals should implement focused preventive measures and provide psychological counseling for these high-risk patients. While numerous studies have highlighted advanced age as a contributing factor to long COVID, the study conducted by Arjun et al. contradicts this notion by suggesting that age does not serve as a significant predictor [21, 22, 25]. Furthermore, our findings suggested that vaccination confers a significant protective influence against the incidence of long COVID, aligning with the outcomes of numerous investigations [22, 26]. Nevertheless, certain studies have presented evidence indicating that there is no significant difference in the development of long COVID between vaccinated patients and unvaccinated patients [21, 27]. The impact of different vaccination levels and post-vaccination duration on the progression of long COVID warrants further investigation to ascertain the efficacy of vaccination in preventing long COVID. In addition, Ayush et al. conducted a study on the determinants influencing the duration of persistent COVID-19 RNA shedding, revealing a correlation between a history of diabetes and chronic kidney disease with prolonged shedding of COVID-19 RNA [28]. The study conducted by Marina et al. demonstrates that individuals who have undergone kidney transplantation exhibit a higher likelihood of persistent shedding of COVID-19 RNA compared to those who have not undergone this procedure [29]. These patients may also have other adverse conditions that pose a higher risk of long COVID, so they should be highly valued to improve their quality of life.

In laboratory tests, leukocytes, lymphocytes, procalcitonin, IL-6 and D-dimer are important indicators for monitoring long COVID risk. Lymphocytes play an important role in the regulation of cellular immunity. It is reported that SARS CoV-2 infection mainly affects T lymphocytes (especially CD4 + T and CD8 + T cells), which means that T lymphocytes may be highly involved in the pathological process of COVID-19 and provide an important means of defense against COVID-19 [30, 31]. The reduction in white blood cells and lymphocytes may suggest a high viral load or compromised immune function in infected individuals, thereby contributing to the development of long COVID. IL-6, a significant inflammatory cytokine, plays a crucial role in certain severe infectious diseases where it triggers a cytokine storm. This storm exacerbates the inflammatory response, initiates the coagulation cascade, promotes disseminated intravascular coagulation (DIC), and potentially results in multi-organ dysfunction [32, 33]. In previous studies, IL-6 and D-dimer have been identified as independent risk factors for the severity of COVID-19 [19, 34, 35]. Besides, procalcitonin is an important diagnostic marker for sepsis [36]. Considering that the above indicators are routine examinations, our new nomogram prediction model can be well applied to hospitals at all levels to identify high-risk populations for long COVID.

As the epidemic progresses, the severe rate and mortality rate of COVID-19 have shown a decline, yet the number of infected people continues to rise, leading to a growing population afflicted with long COVID. Despite the expansion and acceleration of research efforts on long COVID, the current body of knowledge remains inadequate for effective prevention and treatment. To adequately address the long COVID crisis, it is imperative to conduct further research building upon existing knowledge. In view of the above situation, we built a long COVID risk prediction model based on the data of COVID-19 inpatients in a large hospital in central China. The prediction factors used in this model are relatively common, which is more practical and convenient for large-scale screening of high-risk population of long COVID. It may help clinicians to identify those patients with higher long COVID risk early and provide intensive and active treatment to reduce the incidence of long COVID.

This study also has some limitations. Firstly, this is a retrospective single center study that may have some inevitable biases. The outcomes of hospitalized patients may vary depending on their medical resources. Secondly, due to the relaxation of government control measures, many infected individuals have not received nucleic acid or antigen testing, so some patients rely on clinical symptoms for diagnosis, which inevitably leads to misdiagnosis. In addition, the patients in this study were registered at the peak of the COVID-19, when medical resources were scarce, many patients could not be hospitalized in time. In some areas with low epidemic and medical burden, patients with similar conditions may have better outcomes than those receiving treatment in overloaded medical centers. What’s more, in view of the difficulty of follow-up, children were not included in this study as the study population, but the impact of long COVID on this group cannot be ignored [37]. Considering the circumstances, it is advisable to employ the nomogram model established in this study in conjunction with real-world clinical situation.

## Conclusion

In summary, after the Omicron wave, approximately 34.5% of patients reported long COVID symptoms 4–8 weeks after discharge. We established a nomogram model to predict the long COVID risk of hospitalized patients with COVID-19 and proved its relatively good predictive performance. This model has good clinical practicality and is helpful for the clinical management of individuals with high risk of long COVID.

Abbreviations: IL-6, interleukin 6; PCT, Procalcitonin; WBC, white blood cell.

### Electronic supplementary material

Below is the link to the electronic supplementary material.

Supplementary Material 1

Supplementary Material 2

## Data Availability

The datasets used and analyzed during the current study are available from the corresponding author on reasonable request.

## Abbreviations

COVID: Coronavirus disease
AUC: Area under the curve
DCA: Decision curve analysis
PASC: Post acute sequelae of SARS-CoV-2 infection
SD: Standard deviation
IQR: Interquartile range
LASSO: Least absolute shrinkage and selection operator
ROC: Receiver operating characteristic curve
