# Supplementary material for: Development and validation of a prognostic model for assessing long COVID risk following Omicron wave—a large population-based cohort study
Source: Virol J. 2024 May 31;21:123. doi: 10.1186/s12985-024-02400-3 (PMC11140920; doi:10.1186/s12985-024-02400-3)
Supplement: Supplementary file 1 — Supplementary Material 1 [file 12985_2024_2400_MOESM1_ESM.docx]

**Table S1. Additional baseline characteristics of patients in the training and validation cohorts.**

Abbreviations: hs-TnI, High sensitivity Troponin I; CK-MB, creatine kinase-MB; Mb, myoglobin; IL-6, interleukin-6; PCT, Procalcitonin; WBC, white blood cell; MONO, monocyte; RBC, red blood cell; RDW, Red blood cell distribution width; Hct, hematocrit; LYM, lymphocyte; MCV, mean corpuscular volume; MCH, mean corpuscular hemoglobin; MCHC, mean corpuscular hemoglobin concentration; MPV, mean platelet volume; APTT, Activated partial thromboplastin time; TT, thrombin time; INR, international normalized ratio; PT, prothrombin time; PTA, Prothrombin time activity.
